# Supplementary material for: Chromothripsis during telomere crisis is independent of NHEJ, and consistent with a replicative origin
Source: Genome Res. 2019 May;29(5):737–49. doi: 10.1101/gr.240705.118 (PMC6499312; doi:10.1101/gr.240705.118)
Supplement: Supplemental Material [file supp_gr.240705.118_Supplemental_file_1.zip › contigs/annotated_contigs/DB37/contig.2.DB37_length_370_mean_cov_6.83243243243.docx]

**DB37_length_370_mean_cov_6.83243243243**

```
CTCATTATAAAGGGATGCTGAAATGTCCAAGTGTGGAAATGATACGCTTCAGCTCTTATTTTTAAGTCATCCTTATCTCTTTCACTTAT
 >chr16:78668163-78668257 + E=4e-42 p=2e-02
TCCCT|AAAAAAAAAAAA|AAAAATGCTGTTTGACTGGGAGAGCAAGTGGAGCTGTTCAAAGTTCTAGGGACAGCCGGGCGCAGTGGCT
                  >chr16:78668269-78668458 + E=2e-96
CATGCCTGTAATCCCAGCACTTTGGGAGGGCGAGGCGGGCAGATCACGAGGTCAGGAGTTCGAGACCAGCCTGGCCAACATGGTGAAAC

CCCGTCTCTACTAAAAATACAAAAATTAGC|CGGATGTGGTGGTATGCACCTGTAGTCCCAGCTACTCGGGAGGCTGAGGCCTGAGAAT

CACTTGAACCCAGGAGG
```
